# Associations between adverse childhood experiences and cardiometabolic health in later adulthood in Colombia

**DOI:** 10.1136/jech-2024-222234

**Published:** 2025-12-09

**Authors:** Juan Carlos Rivillas-Garcia, Eleanor Margaret Winpenny, Emilie Courtin, Rin Wada, Rachel Neil, Paolo Vineis

**Affiliations:** 1 Department Biostatistics and Epidemiology, MRC Environment and Health, Imperial College London School of Public Health, London, UK; 2 Faculty of Nutrition and Food Sciences, CES University, Medellín, Colombia; 3 Department of Epidemiology and Biostatistics, Imperial College London School of Public Health, London, UK; 4 Department of Health Policy, The London School of Economics and Political Science, London, UK; 5 Johns Hopkins University Bloomberg School of Public Health, Baltimore, Maryland, USA

**Keywords:** CHILD, Life course epidemiology, DIABETES MELLITUS, OBESITY, AGING

## Abstract

**Background:**

Adverse childhood experiences (ACEs) are traumatic events that occur before a child reaches the age of 15 with long-term health consequences, economic costs and intergenerational challenges for society. This study investigated the association between ACEs and cardiometabolic risk (cardiovascular disease (CVD), diabetes, hypertension and obesity) in adulthood.

**Methods:**

We used data from the Survey on Health, Well-Being and Ageing (SABE)-Colombia (n=18 044 adults aged >65). Exposures were defined as single and cumulative ACEs score. Logistic regression, adjusted for demographics and socioeconomic position, was used to investigate associations.

**Results:**

41.3% reported at least one ACE and 4.2% reported four or more. Associations between individual ACEs and outcomes differed by gender. In women, exposure to all ACEs, except childhood migration, was associated with increased odds of CVD, for example, emotional abuse (OR=1.69 (95% CI 1.32 to 2.13)) and poor childhood health status (OR=1.64 (95% CI 1.39 to 1.91)). Among men, these associations were much weaker and often non-statistically significant, except childhood migration that showed increased odds of CVD (OR=1.55 (95% CI 1.09 to 2.15), diabetes (OR=1.55 (95% CI 1.11 to 2.14)) and hypertension (OR=1.40 (95% CI 1.07 to 1.83) in adulthood). A significant association was observed between cumulative ACEs score and odds of CVD, diabetes and hypertension in both men and women. This pattern was not observed for obesity.

**Conclusion:**

The long-term health consequences of ACEs differ by gender. Longitudinal studies are needed to establish causality and identify mediators. Public health interventions should adopt gender-sensitive, holistic approaches integrating biological, environmental, social and behavioural dimensions, and prioritise early-life interventions to address long-term health inequalities.

**WHAT IS ALREADY KNOWN ON THIS TOPIC**

- Evidence from high-income countries (HICs) suggests that early life circumstances (e.g. childhood adversity) are linked to increased risk of cardiometabolic conditions across the lifespan. The influence of ACEs on cardiometabolic health in later adulthood remains understudied in low- and middle-income countries (LMICs), such as Latin American countries. We conducted the present study in a Colombian population to address this knowledge gap.

**WHAT THIS STUDY ADDS**

- We found associations between ACEs and elevated odds of cardiovascular disease (CVD) in women, and CVD, diabetes and hypertension in men. We also found a clear dose-response association between cumulative number of ACEs and an increased odds of CVD risk in women. We identified a connection between early childhood migration due to armed conflict and forced displacement with the odds of CVD, diabetes and hypertension in men but not in women.

**HOW THIS STUDY MIGHT AFFECT RESEARCH, PRACTICE OR POLICY**

- A clear dose-response association suggests that the combination of multiple ACEs may have a cumulative effect on CVD risk, particularly in women. The gender-specific associations found in the study suggest perhaps the need for separate interventions for girls and boys. For instance, incorporating routine screening or prediction tools to identify ACEs histories in adulthood could enable targeted support for individuals at higher risk of developing cardiometabolic conditions. Additionally, understanding the accumulation, severity and combination of ACEs could lead to prevention and intervention strategies that are more tailored to critical periods across the life course.

## Introduction

 Adverse childhood experiences (ACEs) are traumatic events that occur before a child reaches the age of 15. Such adverse experiences can interfere with a person’s health, opportunities, individual’s realisation and stability throughout their lifetime.^1^,^3^ ACEs have been linked to increased risk of cardiovascular disease (CVD),^4^,^6^ diabetes^7 8^ and obesity across the lifespan.^7 9 10^ Childhood emotional maltreatment doubles adult cardiometabolic disease risk.^11^,^13^ Meta-analyses confirm associations of single ACEs^1 9 14^ and cumulative ACEs (with a dose-response relationship) with cardiometabolic outcomes.^15 16^ Women are more likely to be susceptible to the long-term health consequences of ACEs compared with men.^5 6 17 18^

The first ACEs study^19^ categorised ACEs into abuse (physical, emotional and sexual), neglect (physical and emotional) and household dysfunction. While this classification is classic, the field has expanded ACEs to include poverty, familial death, violence, economic hardship and displacement. Nevertheless, research in high-income countries (HICs) often misses adversities prevalent in Latin American contexts. For instance, scarcity of food and migration due to armed conflict are ACEs with higher prevalence in Latin American and Caribbean (LAC) countries. We also highlight the unique patterns and types of ACEs prevalent in LACs, such as community violence and household migration, which differ from those commonly observed in HICs.

ACEs literature suggests that individual ACEs overlook accumulation, co-occurrence, severity and the context of ACEs,^2^ important for understanding long-term health impacts. Early studies demonstrated a dose-response relationship (more ACEs, higher risk).^19^ However, the cumulative number of ACEs or scores, while practical, often assigns equal weight to each adversity, ignoring context (duration, combinations and severity). While cumulative risk of ACEs remains relevant, newer methodologies are emerging, including data-driven clustering,^11 20 21^ severity assessment and examining the perpetrator,^12^ aiming for a more comprehensive understanding of adversity’s complex influence. Recently, latent class analysis (LCA) offers another way to identify subgroups based on ACEs exposure patterns.^22^ This study aims to understand the long-lasting health consequences of ACEs, recognising that ACEs have broader societal implications like increased healthcare costs. The primary research question is as follows: Are ACEs associated with cardiovascular disease, diabetes, hypertension and obesity in later adulthood in a Colombian population and do these associations differ by gender?

## Methods

### Study design and participants selection

We used data from the Health, Well-Being and Ageing Study (SABE-Colombia, first wave). This study used a multistage random cluster sampling method to select 240 municipalities. All older adults eligible within these municipalities were chosen at random from the master sample of the Ministry of Health and Social Protection. 36 153 adults aged 65 and older were included in the sample; of these, 23 694 completed the survey questionnaires, giving a national response rate of 66%. In this study, we included 18 044 participants who had complete demographic, exposure and health outcomes data. Each participant provided written informed consent, and a local ethics committee approved the study (University of Caldas and University of Valle). Further information on the representativeness of the sample, survey implementation, data collection, quality control and ethical approval is provided elsewhere.^23 24^ Online supplemental figure 1 provides the number and definition of cases in the study population.

### Outcomes

The primary outcome variables are binary (disease=Yes or No). SABE-Colombia collected data by interviewing older persons and it provides data on outcomes from self-reported diagnoses of cardiovascular disease, hypertension and diabetes. CVD includes self-reported episodes of heart attack, angina and thrombosis. Body-mass index (BMI) was calculated based on nurse-measured height and weight (kg / (height (m))^2^) and used an established cut-off to indicate obesity (BMI>=30).

### Exposures

Data on ACEs that occurred before the age of 15 were obtained from the SABE-Colombia questionnaire and covered five questions related to poor health status, scarcity of food during childhood, childhood maltreatment, emotional abuse and childhood migration due to armed conflict and forced displacement. Participants in SABE-Colombia were asked whether they have been exposed to specific ACEs. All responses were dichotomised to having been exposed or non-exposed to these single ACEs (‘yes/one or more times’ or ‘no/never’). This migration variable was filtered to refer solely to forced displacement and does not include voluntary moves undertaken for economic reasons. Single ACEs and cumulative childhood adversity were used as exposures. Online supplemental table 1 presents the definition of individual and cumulative ACEs, and online supplemental table 2 presents the correlation matrix of ACEs.

### Covariates

During the interview, participants were asked to report their age, gender (female and male) and ethnic group. The response categories given for ethnic groups were Mestizo (mixed European and Amerindian heritage), white, Afro-Colombian (including Raizales, Palenqueros and Afro-descended populations), Indigenous and other.

A Direct Acyclic Graph (DAG) was used to describe the role of confounders in the relationship between ACEs exposures and adult cardiometabolic outcomes. We identified age, ethnicity and low childhood socio-economic position (SEP) as confounders (online supplemental figure 2).

### Statistical analysis

We first explored the correlations across ACEs. We constructed a tetrachoric correlation matrix calculating Rho coefficients to indicate the strength of association between single adversities. Pearson’s χ^2^ tests were used to compare differences between categorical variables. Second, to assess ACE exposure, we employed three established methods.^2 12 25^ We examined each ACE individually and calculated an ACEs score^2^ by summing the number of single adversities as ACEs 0, ACEs 1, ACEs 2, ACEs 3 or ACE 4+.

To examine the associations between each of the ACE exposures and four diseases (cardiovascular disease, diabetes, hypertension and obesity), we conducted logistic regression analyses stratified by gender. We controlled for potential confounding variables to isolate the effect of ACEs on cardiometabolic health outcomes and ensure the robustness of the findings. The confounders include sociodemographic factors such as age and ethnicity and low childhood socioeconomic position. Age may also influence childhood socioeconomic position (SEP) and is a critical confounder of the risk of cardiometabolic disease (along a path that does not include the exposure). Ethnicity was included as an important confounder due to the historical and ongoing social inequalities and systematic discrimination faced by various ethnic groups in Colombia, which may influence ACE experiences, and the known links between ethnicity and cardiometabolic health.^26^

Childhood SEP refers to a child’s access to family resources, typically measured through indicators such as maternal education, paternal occupation, paternal education levels and overall family socioeconomic position.^27 28^ Finally, childhood SEP was included as a confounder because it is closely linked to both ACEs and cardiometabolic risk. Individuals from the most disadvantaged settings are more likely to experience ACEs and face increased risk factors for cardiometabolic risk. By controlling for low childhood SEP, we can more accurately isolate the independent contribution of ACEs to cardiometabolic disease in adulthood. Lifestyle risk factors such as smoking, physical inactivity, alcohol intake and medication and low adult socioeconomic position are not included as confounders since they occur after exposure to ACEs.^29^

We fitted two sets of models for each ACEs operationalisation: a crude model and a model adjusted for age, ethnicity and low childhood SEP. Additionally, to test the trend with the number of ACEs, we performed a Cochran Armitage test.^30^ R^2^ Tjur measure was used to evaluate model fit. All analyses were conducted using R Studio version 2021.09.1.

## Results

The descriptive characteristics of the study population are reported in table 1. The study population consisted predominantly of women (56%) with an average age of 69.1 (SD 7.05) years and was primarily Mestizo (43%). The two most reported ACEs were scarcity of food (26.6%).

**Table 1:** Baseline characteristics, ACEs and disease of the study participants by gender

Characteristics/ Categories	All	Gender		P value
		Women	Men	
N	18 044	10 101	7943	
Sociodemographic characteristics				
Mean age, years* (SD)	69.1 (7.05)	68.8 (6.98)	69.4 (7.13)	<0.001
Median age (Min, Max)	68.0 (60.0, 101)	67.0 (60.0, 101)	68.0 (60.0, 100)	
Ethnicity, n (%)				<0.001
Afro Colombian	2125 (11.8%)	1150 (11.4%)	975 (12.3%)	
Indigenous	1438 (8.0%)	687 (6.8%)	751 (9.5%)	
Mestizo	7757 (43%)	4262 (42.2%)	3495 (44%)	
Other	1772 (9.8%)	1105 (9.9%)	667 (8.4%)	
White	4952 (27.4%)	2897 (28.7%)	2055 (25.9%)	
Low childhood SEP (Yes)	10 728 (59.5%)	7924 (78.4%)	6587 (82.9%)	<0.001
Single adversity (age<15 years, Yes)				
Emotional abuse, n (%)	792 (4.4%)	467 (4.6%)	325 (4.1%)	0.090
Childhood maltreatment, n (%)	2984 (16.5%)	1792 (17.7%)	1192 (15%)	<0.001
Childhood migration,† n (%)	525 (2.9%)	291 (2.9%)	234 (2.9%)	0.831
Scarcity of food, n (%)	4805 (26.6%)	2537 (25.1%)	2268 (28.6%)	<0.001
Childhood poor health status, n (%)	2168 (12%)	1131 (11.2%)	1037 (13.1%)	<0.001
Cumulative childhood adversity (age<15 years): ACEs score,‡ n (%)				0.013
ACEs 0	4871 (27%)	2656 (26.3%)	2144 (27%)	
ACEs 1	7452 (41.3%)	4222 (41.8%)	3280 (41.3%)	
ACEs 2	2959 (16.4%)	2141 (16.2%)	1302 (16.4%)	
ACEs 3	2020 (11.2%)	1161 (11.5%)	889 (11.2%)	
ACEs 4+	757 (4.2%)	424 (4.2%)	333 (4.2%)	
Adult cardiometabolic outcomes (age>65 years old, Yes)				
Cardiovascular disease, n (%)	2331 (12.9%)	1355 (13.4%)	976 (12.3%)	0.027
Diabetes, n (%)	2925 (16.2%)	1846 (18.3%)	1079 (13.6%)	<0.001
Hypertension, n (%)	9374 (52%)	5818 (57.6%)	3556 (44.8%)	<0.001
Obesity, n (%)	6021 (33.4%)	3877 (38.4%)	2144 (27%)	<0.001

* Data is displayed as n (%) for categorical variables, mean (SD) and median for age.

† Between 0 and 15 years old.

‡ Cumulative ACEs score range 0–4 and more.

ACE, adverse childhood experiences; SEP, socio-economic position .

Women reported more ACEs and had higher rates of emotional abuse and childhood maltreatment compared with men, while men reported more poor health status and scarcity of food. No gender differences were observed for childhood migration. The study population showed a high prevalence of hypertension (52%) and obesity (33.4%).

Table 2 details sample characteristics by cumulative ACEs exposure. As the ACEs score increases, the prevalence of CVD, diabetes, hypertension and obesity generally tends to increase. This is most evident among those individuals exposed to four or more ACEs, which consistently shows higher percentages for these conditions compared with the ACEs zero category. The prevalence of hypertension slightly rises from 52.5% in ACEs 0 to 53.5% in ACEs 4+. In obesity, the prevalence rises from 23% in ACEs zero to 28.2% in ACEs 4+. The prevalence of diabetes goes from 15.7% in ACEs zero to 15.5% in ACEs 4+. Although not a linear increase, the two and three ACEs groups show higher percentages than ACEs 0. While the prevalence of cardiovascular disease is 12.9% in ACEs 0 and 19% in ACEs 4+. This suggests that experiencing more ACEs puts individuals at a higher risk of developing cardiometabolic diseases later in life.

**Table 2:** Sample characteristics and prevalence of cardiometabolic disease by cumulative ACEs exposure

Characteristic	Cumulative childhood adversity (age<15 years): ACEs score				
	ACEs 0	ACEs 1	ACEs 2	ACEs 3	ACEs 4+
	4871 (27%)	7452 (41.3%)	2959 (16.4%)	2020 (11.2%)	757 (4.2%)
Age means	69.3	69.4	68.7	67	68
Sex					
Women	2839 (58.3%)	4515 (60.6%)	1757 (59.4%)	1236 (61.2%)	458 (60.6%)
Men	2031 (41.7%)	2936 (39.4%)	1201 (40.6%)	783 (38.8%)	298 (39.4%)
Race/ethnicity					
Afro Colombian	467 (9.6%)	625 (8.4%)	272 (9.2%)	159 (7.9%)	26 (3.5%)
Indigenous	267 (5.5%)	462 (6.2%)	213 (7.2%)	133 (6.6%)	85 (11.3%)
Mestizo	2581 (53%)	3882 (52.1%)	1479 (50%)	1012 (50.1%)	383 (50.7%)
Other	409 (8.4%)	700 (9.4%)	287 (9.7%)	191 (9.5%)	80 (10.6%)
White	1139 (23.4%)	1773 (23.8%)	704 (23.8%)	523 (25.9%)	180 (23.9%)
Cardiometabolic disease in adulthood					
Cardiovascular disease	628 (12.9%)	1020 (13.7%)	396 (13.4%)	250 (12.4%)	143 (19%)
Diabetes	764 (15.7%)	798 (16.8%)	511 (17.3%)	377 (18.7%)	117 (15.5%)
Hypertension	2557 (52.5%)	4113 (55.2%)	1659 (56.1%)	1119 (55.4%)	404 (53.5%)
Obesity	1120 (23%)	1937 (26%)	807 (27.3%)	523 (25.9%)	213 (28.2%

Note: Cumulative childhood adversity (age <15 years): ACEs score is based on five questions related to poor health status, scarcity of food during childhood, childhood maltreatment, emotional abuse and childhood migration due to armed conflict and forced displacement.

ACE, adverse childhood experiences.

We found a significant interaction between ACEs and gender on the odds of cardiometabolic risk and therefore conducted all analyses stratified by gender (table 3). Women exposed to specific ACEs (except childhood migration) had higher odds of cardiovascular disease than women without these experiences (emotional abuse OR=1.68 (95% CI 1.34 to 2.12; p<0.001)), poor childhood health status (OR=1.66 (95% CI 1.41 to 1.94; p<0.001), childhood maltreatment (OR=1.55 (95% CI 1.35 to 1.79; p<0.001)) and scarcity of food (OR=1.44 (95% CI: 1.26 to 1.64; p<0.001)). Men generally had lower odds of CVD for the same ACEs, except for childhood migration that showed increased odds of diabetes (OR=1.60 (95% CI 1.14 to 2.20; p=0.005)), cardiovascular disease (OR=1.55 (95% CI 1.09 to 2.15; p=0.012)), and hypertension (OR=1.43 (95% CI 1.10 to 1.86; p=0.008)). Adjustments for demographic factors and childhood SEP minimally changed ORs. Online supplemental table 3 reports unadjusted and adjusted models for overall population.

**Table 3:** The association of individual ACEs with self-reported cardiometabolic health outcomes in adulthood

ACEs type/ outcomes	Women				Men			
	CVD	Diabetes	Hypertension	Obesity	CVD	Diabetes	Hypertension	Obesity
	ORs (95% CI)	ORs (95% CI)	ORs (95% CI)	ORs (95% CI)	ORs (95% CI)	ORs (95% CI)	ORs (95% CI)	ORs (95% CI)
Emotional abuse (Reference=non-exposed)								
Unadjusted model	1.65 (1.30 to 2.08)***	1.09 (0.85 to 1.37)	1.17 (0.97 to 1.42)	1.22 (1.01 to 1.48)*	1.22 (0.88 to 1.65)	0.94 (0.67 to 1.30)	1.15 (0.92 to 1.43)	1.19 (0.93 to 1.52)
Age and ethnicity-adjusted model	1.71 (1.34 to 2.16)***	1.11 (0.87 to 1.39)	1.24 (1.02 to 1.50)*	1.20 (0.99 to 1.45)	1.33 (0.95 to 1.81)	0.99 (0.70 to 1.36)	1.20 (0.96 to 1.51)	1.19 (0.95 to 1.49)
Multivariable model	1.68 (1.32 to 2.12)***	1.09 (0.86 to 1.37)	1.20 (0.98 to 1.45)	1.18 (0.98 to 1.43)	1.32 (0.95 to 1.80)	0.98 (0.69 to 1.35)	1.21 (0.96 to 1.52)	1.20 (0.94 to 1.52)
Childhood maltreatment (Reference=non-exposed)								
Unadjusted model	1.47 (1.28 to 1.69)***	1.10 (0.99 to 1.28)	0.99 (0.90 to 1.10)	1.03 (0.93 to 1.14)	1.18 (0.98 to 1.40)*	1.15 (0.96 to 1.36)	1.00 (0.89 to 1.14)	1.14 (0.99 to 1.30)
Age and ethnicity-adjusted model	1.58 (1.37 to 1.81)***	1.12 (0.98 to 1.27)	1.07 (0.97 to 1.19)	0.98 (0.88 to 1.09)	1.26 (1.05 to 1.51)*	1.16 (0.97 to 1.37)	1.07 (0.94 to 1.21)	1.12 (0.97 to 1.28)
Multivariable model†	1.55 (1.35 to 1.79)***	1.11 (0.97 to 1.30)	1.03 (0.93 to 1.15)	0.96 (0.86 to 1.07)	1.24 (1.03 to 1.49)*	1.13 (0.94 to 1.34)*	1.05 (0.92 to 1.19)	1.10 (0.96 to 1.26)
Childhood migration (Reference=non-exposed)								
Unadjusted model	1.06 (0.75 to 1.47)	1.07 (0.79 to 1.42)	1.04 (0.82 to 1.31)	1.08 (0.85 to 1.37)	1.64 (1.15 to 2.27)**	1.63 (1.16 to 2.23)**	1.48 (1.14 to 1.93)**	1.21 (0.91 to 1.60)
Age and ethnicity-adjusted model	1.01 (0.71 to 1.40)	1.06 (0.78 to 1.41)	0.98 (0.77 to 1.24)	1.12 (0.88 to 1.42)	1.55 (1.09 to 2.16)*	1.61 (1.15 to 2.22)**	1.44 (1.10 to 1.88)**	1.22 (0.91 to 1.61)
Multivariable model^(a)^	1.00 (0.70 to 1.38)	1.05 (0.77 to 1.40)	0.96 (0.75 to 1.22)	1.11 (0.87 to 1.41)	1.55 (1.09 to 2.15)*	1.60 (1.14 to 2.20)**	1.43 (1.10 to 1.86)**	1.21 (0.91 to 1.60)
Scarcity of food (Reference=non-exposed)								
Unadjusted model	1.40 (1.24 to 1.59)***	1.13 (1.01 to 1.27)	1.08 (0.98 to 1.18)*	0.97 (0.88 to 1.06)	1.16 (0.98 to 1.34)*	1.04 (0.90 to 1.19)	1.11 (1.00 to 1.22)	0.94 (0.84 to 1.04)
Age and ethnicity-adjusted model	1.46 (1.29 to 1.66)***	1.15 (1.02 to 1.29)	1.12 (1.02 to 1.23)	0.93 (0.85 to 1.02)	1.21 (1.04 to 1.40)*	1.05 (0.91 to 1.21)	1.13 (1.03 to 1.25)*	0.92 (0.83 to 1.03)
Multivariable model^(a)^	1.44 (1.26 to 1.64)***	1.12 (1.00 to 1.27)	1.06 (0.96 to 1.16)	0.90 (0.82 to 0.99)	1.19 (1.02 to 1.38)*	1.01 (0.87 to 1.16)	1.10 (0.99 to 1.22)	0.89 (0.80 to 1.00)
Childhood poor health status (Reference=non-exposed)								
Unadjusted model	1.64 (1.40 to 1.89)***	1.10 (0.94 to 1.28)	1.00 (0.89 to 1.13)	0.99 (0.86 to 1.10)	1.11 (0.91 to 1.33)	1.20 (1.01 to 1.43)	1.14 (1.01 to 1.30)*	0.94 (0.81 to 1.08)
Age and ethnicity-adjusted model	1.67 (1.42 to 1.96)***	1.10 (0.94 to 1.29)	1.00 (0.89 to 1.14)	0.97 (0.86 to 1.11)	1.16 (0.95 to 1.40	1.21 (1.00 to 1.45)*	1.17 (1.02 to 1.33)*	0.93 (0.80 to 1.08)
Multivariable model^(a)^	1.66 (1.41 to 1.94)***	1.09 (0.93 to 1.27)	0.99 (0.87 to 1.13)	0.97 (0.85 to 1.10)	1.15 (0.94 to 1.39)	1.20 (1.00 to 1.44)	1.16 (1.03 to 1.35)*	0.93 (0.80 to 1.07)

*p < 0.05; **p = 0.01; ***p < 0.001.

†Multivariable model: age and ethnicity-adjusted model + low childhood SEP (yes, no).

ACE, adverse childhood experiences; CVD, cardiovascular disease.

**Figure 1:**
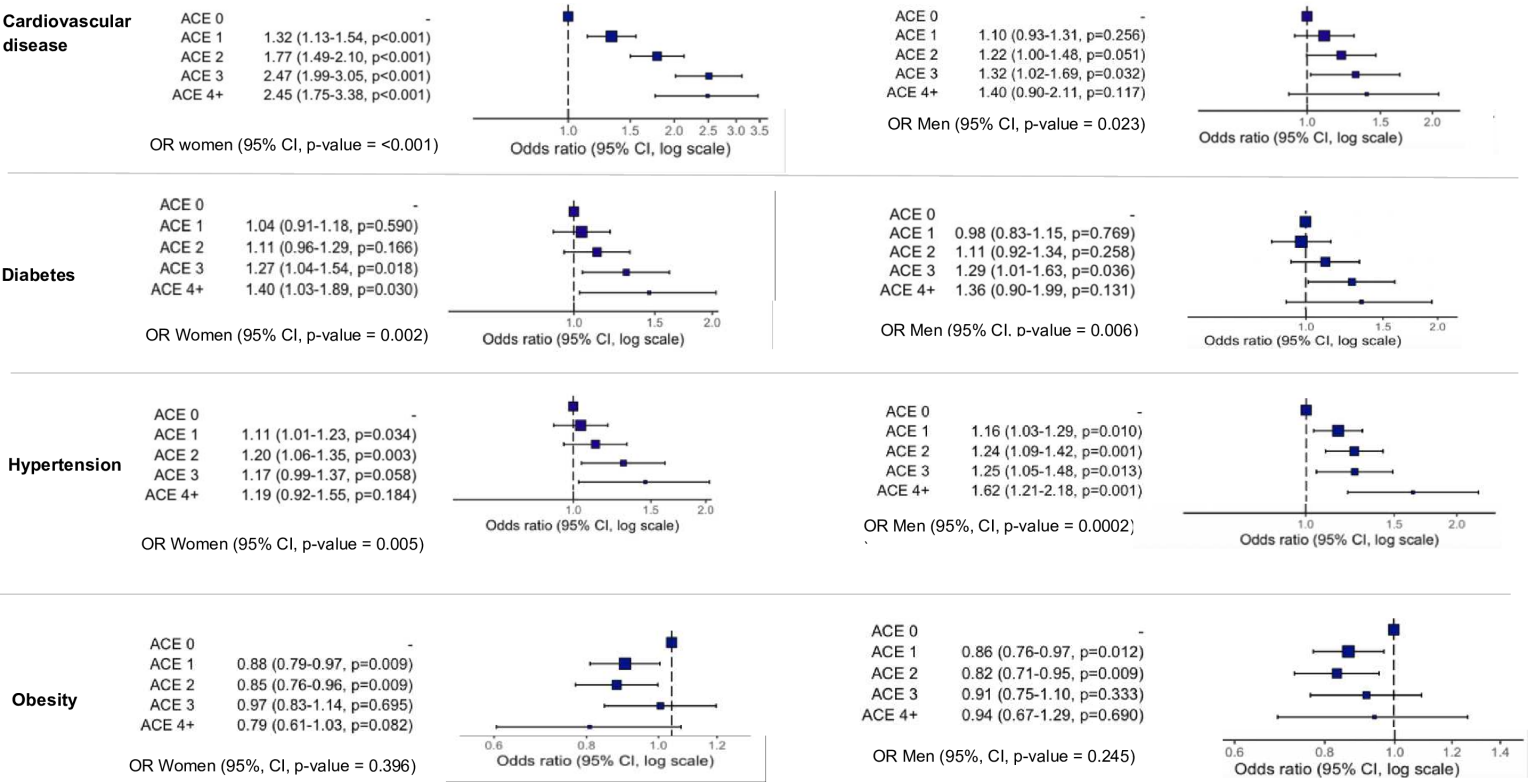
Association of the cumulative adverse childhood experiences with the odds of cardiometabolic disease in adulthood by gender. Notes for interpretation: Adjusted odds ratios for demographic factors (age and ethnicity), and low childhood SEP. ACE, adverse childhood experiences; ACE 0 (reference), not exposed to ACE; ACE 1, exposed to one ACE; ACE 2, exposed to two ACE; ACE 3, exposed to three ACEs; ACEs 4+, exposed to 4 or more ACE. OR were calculated from logistic regression models. Midpoints of the lines represent the ORs, and error bars show the 95% CI, 95% CIs. ACE, adverse childhood experiences; SEP, socio-economic position

Table 4 reports the results of the association between cumulative ACEs with health outcomes. Figure 1 illustrates the dose-response relationship between the cumulative ACEs and cardiometabolic risk. Each additional ACE is associated with increased odds of CVD risk in women and hypertension in men. Adjustments for demographic factors and childhood SEP did not substantially change this gradient. A significant interaction was observed between cumulative ACEs and CVD by gender (p<0.001). A clear dose-response association between ACEs score and higher odds of CVD risk was observed, with women experiencing a particularly high OR with three or more ACEs (one, OR=1.32 (95% CI 1.13 to 1.55; p<0.001); two, OR=1.77 (95% CI1.50 to 2.11; p<0.001); three, OR=2.48 (2.01–3.05; p<0.001) and four or more, OR=2.46 (95% CI 1.76 to 3.39; p<0.001); *P* trend<0.001). While obesity showed a weak association with ACEs in both genders, diabetes showed a stronger association in women with multiple ACEs. Men with three ACEs showed higher odds of CVD and hypertension (online supplemental table 4).

**Table 4:** Association between cumulative ACEs and cardiometabolic disease in adulthood by gender

Exposures/ outcomes	Women				Men			
	Cardiovascular disease	Diabetes	Hypertension	Obesity	Cardiovascular disease	Diabetes	Hypertension	Obesity
	ORs (95% CI)	ORs (95% CI)	ORs (95% CI)	ORs (95% CI)	ORs (95% CI)	ORs (95% CI)	ORs (95% CI)	ORs (95% CI)
Cumulative childhood adversity (age<15 years): ACEs score: Unadjusted model (Reference=ACEs 0)								
ACEs 1	1.31 (1.13 to 1.53)***	1.03 (0.91 to 1.17)	1.11 (1.01 to 1.23)*	0.88 (0.79 to 0.97)**	1.08 (0.92 to 1.28)	0.98 (0.83 to 1.15)	1.14 (1.02 to 1.27)*	0.87 (0.77 to 0.98)*
ACEs 2	1.72 (1.45 to 2.04)***	1.11 (0.96 to 1.29)	1.19 (1.06 to 1.33)*	0.89 (0.79 to 1.00)*	1.18 (0.97 to 1.43)	1.13 (0.94 to 1.35)	1.21 (1.07 to 1.38)**	0.85 (0.73 to 0.98)*
ACEs 3	2.35 (1.91 to 2.89)***	1.27 (1.05 to 1.54)	1.16 (1.00 to 1.36)	1.03 (0.88 to 1.20)	1.26 (0.98 to 1.61)	1.31 (1.04 to 1.65)	1.20 (1.02 to 1.42)*	0.95 (0.79 to 1.14)
ACEs 4+	2.28 (1.64 to 3.14)***	1.40 (1.03 to 1.88)	1.17 (0.91 to 1.51)	0.86 (0.67 to 1.12)	1.29 (0.83 to 1.93)	1.34 (0.89 to 1.95)	1.53 (1.15 to 2.05)**	0.97 (0.70 to 1.33)
Cumulative childhood adversity (age<15 years): Multivariable model^a^: Adjusted model for demographic factors and childhood socioeconomic position (Reference=ACEs 0)								
ACEs 1	1.32 (1.13 to 1.54)***	1.04 (0.91 to 1.18)	1.11 (1.01 to 1.23)*	0.88 (0.79 to 0.97)**	1.10 (0.93 to 1.31)	0.98 (0.83 to 1.15)	1.16 (1.03 to 1.29)*	0.86 (0.76 to 0.97)*
ACEs 2	1.77 (1.49 to 2.10)***	1.11 (0.96 to 1.29)	1.20 (1.06 to 1.35)**	0.85 (0.76 to 0.96)**	1.22 (1.00 to 1.48)	1.11 (0.92 to 1.34)	1.24 (1.09 to 1.42)***	0.82 (0.71 to 0.95)**
ACEs 3	2.47 (1.99 to 3.05)***	1.27 (1.04 to 1.54)*	1.17 (0.99 to 1.37)	0.97 (0.83 to 1.14)	1.32 (1.02 to 1.69)*	1.29 (1.01 to 1.63)*	1.25 (1.05 to 1.48)*	0.91 (0.75 to 1.10)
ACEs 4+	2.45 (1.75 to 3.38)***	1.40 (1.03 to 1.89)*	1.28 (0.92 to 1.55)	0.79 (0.61 to 0.82)	1.40 (0.90 to 2.11)	1.36 (0.90 to 1.99)	1.62 (1.21 to 2.18)**	0.94 (0.67 to 1.29)
P for trend	**<0.001**	**0.002**	**0.005**	0.396	**0.023**	**0.006**	**0.0002**	0.245

*p < 0.05; **p= 0.01; ***p< 0.001.

ACE, adverse childhood experiences.

## Discussion

A significant proportion of the participants (71%) reported experiencing at least one ACE, with a higher prevalence in women and mestizo older adults. The prevalence of ACEs in the SABE-Colombia population (28.6% with zero ACEs and 2.6% reporting four or more ACEs) aligns with a previous systematic review,^16^ reporting a wide range in ACE prevalence across populations: on average 57% of participants across all studies reported at least one ACE and 13% reported at least four. Our study found gender disparities in the association between ACEs and the odds of cardiometabolic risk (CVD, diabetes and hypertension) in later adulthood in the Colombian population. It supports previous research.^31^ However, our study diverges from previous findings in the UK^2^ that found no gender differences, highlighting context-specific impacts.

In our study, women exposed to emotional abuse, scarcity of food, childhood poor health status and childhood maltreatment had higher odds of CVD than men. Childhood maltreatment was associated with a significant increase in cardiovascular disease for women only in other studies from Germany, The Netherlands, the UK and the USA.^31 32^ This evidence supports the lasting health consequences of this specific early life stressor on women’s adult health. The observed higher odds of cardiovascular disease among women compared with men with similar ACEs exposure may be attributed to a complex interplay of biological, socioeconomic and behavioural factors and their cumulative consequences. Additionally, complex interactions of biological factors, exacerbated by hormonal changes during menopause, can increase the odds of cardiovascular risk in women.^33^ The development of resilience and coping mechanisms among women to reduce the adverse effects of ACEs may be a potential explanation. Furthermore, protective factors against CVD resulting from genetic factors may be involved in various types of ACEs at individual levels.

In this study, we found that only among men, migration before age 15 due to stressful circumstances was associated with higher odds of heart disease, stroke, diabetes and elevated blood pressure, whereas women exposed to similar migratory experiences showed no increase in odds of adverse cardiometabolic health outcomes. Our findings should be interpreted within the context of Colombia’s prolonged internal conflict and large-scale displacement during the mid-20th^th^ century. Between approximately 1955 and 1965, the country experienced intense violence related to the emergency of insurgent groups, producing one of Latin American’s earliest and largest waves of internal migration.^34^ Rural residents were forcibly displaced or migrated to urban areas, often under severe psychological and socioeconomic stress. This population movement reshaped Colombia’s demographic landscape, with rapid urbanisation, family disruption and loss of livelihoods disproportionately affecting rural men engaged in agriculture.

For the SABE-Colombia study, many participants would have spent their earlier childhood or adolescence during this turbulent and stressful period, meaning that early-life migration likely coincided with nutritional deprivation, educational interruption and chronic stress exposure factors that were plausibly linked to later cardiometabolic risk. The absence of similar association among women may reflect gendered differences in Colombian migration experiences, exposure intensity or post-migration roles, as women may have migrated primarily within family units and faced different adaptive demands. This interpretation aligns with prior research linking early-life migration from China to Hong Kong to adverse cardiovascular and metabolic outcomes in adulthood.^35^

In another context, Schooling *et al*.’s findings linked migration during the first 7 years of life to the development of hypertension and heart disease in men (migration at ages 0–7 OR=3.17, 95% CI 1.70 to 5.91). Women who migrated before age 25 did have higher risks, but not statistically significantly so. Moreover, although forced migration is a stressful circumstance that may have led to improved living conditions and access to healthcare services post-migration, improving cardiovascular health. Also, there may be other unmeasured confounders that influence the association between ACEs and CVD not captured in the models. This selective migration effect, coupled with potential protective parental SEP, could play an important role.

Our analysis suggests that hypertension may not follow a strictly linear pattern, with different ACE types showing varying associations, consistent with prior meta-analyses.^14 36^ A clear dose-response association was found between cumulative ACEs and higher odds of CVD and diabetes, with women being more vulnerable than men: odds of CVD associated with ACE exposure in women were more than double those seen in men. This gender difference aligns with existing research,^437^,^39^ which has proposed that biological stress responses, differences in coping mechanisms and gendered social roles may amplify the long-term cardiometabolic impacts of childhood adversity in women.

Women with one or two ACEs in our sample had lower odds of obesity compared with those reporting no ACEs, contrary to existing literature suggesting a positive association between ACEs and obesity risk.^9 16^ Several plausible explanations may help to understand this unexpected finding. First, survival bias could play a role in older cohorts if women with higher ACE exposure and obesity-related morbidity were less likely to survive to participate in later-life surveys. Second, energy expenditure and nutritional deprivation during periods of conflict or contextual factors relevant to some cohorts may be attenuating the association between ACEs and later adiposity. Third, the socioeconomic position and related behavioural factors, such as dietary habits, physical activity and access to healthcare, may mediate or modify these associations, potentially obscuring expected patterns. Finally, measurement limitations or differences in the timing and type of ACE exposure may also influence observed associations.

These findings require longitudinal data to better understand the pathways linking ACEs, gender and SEP factors to adult cardiometabolic outcomes. Future work incorporating life-course trajectories, mediating psychological and behavioural factors, and cohort-specific contexts will be key to understanding these complex and, at times, counterintuitive associations.

### Strengths and limitations

The main strength of this study is that it is one of the first studies examining associations between ACEs and cardiometabolic health in a Latin American population. It is also one of the first studies to include childhood migration as an ACE, taking advantage of the particular context of this cohort. Additionally, the representative sample of older adults enhances the generalisability of our findings. Furthermore, this study utilised the ACEs questionnaire from one of the first Ageing surveys in the Latin American and Caribbean (LAC) region, highlighting the value of early life epidemiology approaches.

This study examining retrospective ACEs and cardiometabolic outcomes faces limitations. Causal inference remains difficult and future longitudinal studies with broader ACEs assessment are needed to clarify these associations. Recall bias, particularly among older adults normalising childhood abuse, may influence gender-specific associations. The absence of data on crucial ACEs like parental mental illness, maternal smoking and parental unemployment likely underestimates exposure. Exclusion of older adults with cognitive impairment may restrict generalisability and introduce selection bias, as this group may have different ACE profiles.

## Conclusion

This study reveals gender-specific patterns in the association between childhood adversity and cardiometabolic risk, particularly highlighting lasting consequences for women’s cardiometabolic health. Early childhood migration due to armed conflict and forced displacement was demonstrated to have an important role in increased risk of CVD, diabetes and hypertension in men but not among women. Traditional ACE measures such as examining individual ACEs as well as cumulative childhood adversity measured using an ACEs score should continue to be used. In this study, the derivation of latent ACE classes did not improve the findings. Our results highlight the need for longitudinal research to identify mediators and moderators, such as genetic, social and environmental factors, to enable further understanding of the causal pathways underlying these associations. Findings from our study suggest that public health interventions should adopt gender-sensitive, holistic approaches integrating biological, environmental, social and behavioural dimensions, and prioritise early-life interventions to address long-term health inequalities, particularly for girls. Further work is also needed to increase awareness about the high cost of childhood adversities for healthcare systems in the long term.

## Supplementary material

10.1136/jech-2024-222234 online supplemental file 1

## Data Availability

Data may be obtained from a third party and are not publicly available.
