# Supplementary material for: Associations between adverse childhood experiences and cardiometabolic health in later adulthood in Colombia
Source: J Epidemiol Community Health. 2025 Dec 9;80(5):e222234. doi: 10.1136/jech-2024-222234 (PMC13151427; doi:10.1136/jech-2024-222234)
Supplement: online supplemental file 1 [file jech-80-5-s001.pdf]

Supplementary material

Associations between Adverse Childhood Experiences and Cardiometabolic Health in Later Adulthood in Colombia

**Supplementary Figure 1**	Selection of participants and analytical sample.
**Supplementary Figure 2**	The total causal effect of adverse childhood experiences on health outcomes and the role of confounders in this interrelationship.
**Supplementary Table 1**	Definition of individual adverse childhood experiences and cumulative childhood adversity.
**Supplementary Table 2**	Correlation of adverse childhood adversities in the SABE-Colombia-2015
**Supplementary Table 3**	Unadjusted and adjusted odds ratio of individual adverse childhood experiences (ACEs) and cardiometabolic disease in later adulthood
**Supplementary Table 4**	Associations of cumulative childhood adversity and cardiometabolic disease in later adulthood

Supplementary Figure 1. Selection of participants and analytical sample.

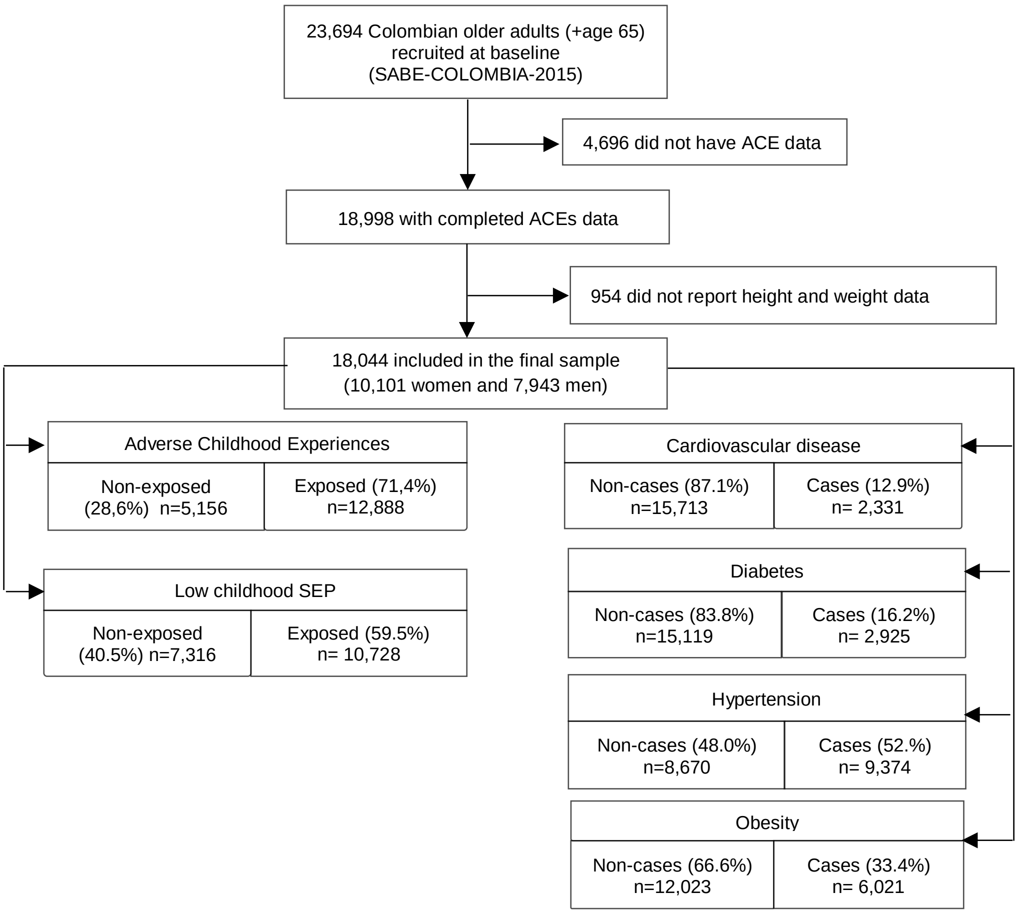

Supplementary Figure 2. The total causal effect of adverse childhood experiences on health outcomes and the role of confounders in this interrelationship.

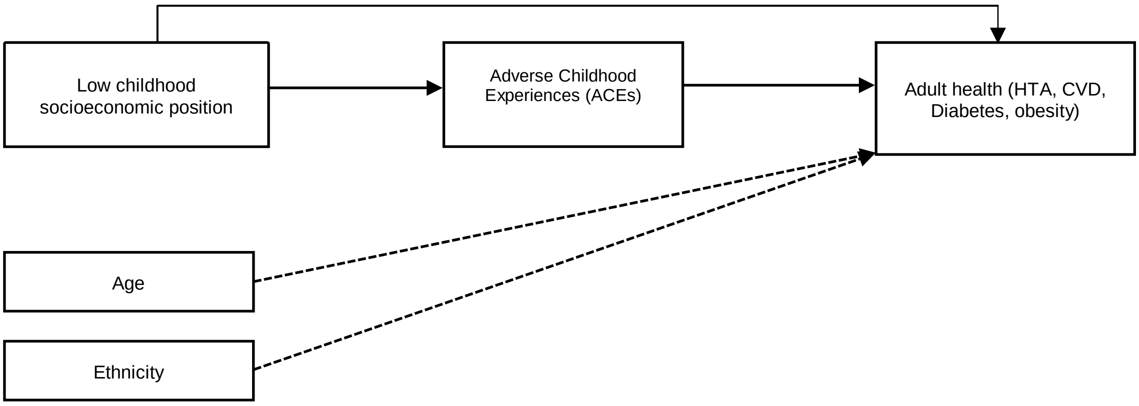

Supplementary Table 1. Definition of individual adverse childhood experiences and cumulative childhood adversity.

ACEs assessment	Description	How ACE was categorized
Emotional abuse	Positive response to 'victim of emotional abuse during childhood'	Yes/No
Childhood maltreatment	Positive response to ever having 'experienced domestic violence' and stating age experienced was < 15 years.	Yes/No
Childhood migration	Positive response to ever having 'experience being displaced due to armed conflict' and stating age was < 15 years.	Yes/No
Scarcity of food	Positive response to ever having 'experienced being starving and stating experienced age was < 15 years.	Yes/No
Poor childhood health status	Positive response to ever having 'sick and stating age was < 15 years.	Yes/No
Cumulative childhood adversity (age <15 years): ACEs score	Derived from the 6-item individual adverse childhood experiences (continuous variable)	From ACE 0 to four or more ACEs
Any ACEs	Derived from the 6-item individual adverse childhood experiences (categorical variable)	Zero ACE/At least one ACE

Supplementary Table 2. Correlation of adverse childhood adversities in the SABE-Colombia-2015.

This table shows weak positive correlations between single ACEs. Participants reporting domestic violence also often reported scarcity of food (r=0.36, 29%), and poor childhood health status was commonly associated with scarcity of food (r=0.30, 44%).

Adversity	Emotional abuse	Scarcity of food	Poor childhood health	Childhood maltreatment	Childhood migration
N	792	4,805	2,168	2,984	525
Emotional abuse		6.95% (0.20)	7.56% (0.17)	8.34% (0.24)	5.71% (0.06)
Scarcity of food	42.2% (0.20)		44.7% (0.30)	46.7% (0.36)	33.5% (0.09)
Poor childhood health	20.7% (0.17)	20.2% (0.30)		18.8% (0.20)	17.1% (0.10)
Childhood maltreatment	31.4% (0.24)	29.0% (0.36)	25.8% (0.20)		25.5% (0.14)
Childhood migration	3.79% (0.06)	3.66% (0.09)	4.15% (0.10)	4.49% (0.14)	

Note for interpretation: The percentages (%) represent the overall prevalence of co-reporting adversities. The value in parenthesis indicates correlation coefficient (Rho). Each percentage reflect the proportion of individuals in the total sample for each column who also reported the corresponding ACEs in the row.

Supplementary Table 3. Unadjusted and adjusted odds ratio of individual adverse childhood experiences (ACEs) and cardiometabolic disease in later adulthood.

**Variable**	**Hypertension**				**Diabetes**				**Cardiovascular disease**				**Obesity**			
	**Unadjusted**		**Adjusted**		**Unadjusted**		**Adjusted**		**Unadjusted**		**Adjusted**		**Unadjusted**		**Adjusted**	
	Odd ratios (95% CI)	*P value*	Odd ratios (95% CI)	*P value*	Odd ratios (95% CI)	*P value*	Odd ratios (95% CI)	P value	Odd ratios (95% CI)	*P value*	Odd ratios (95% CI)	P value	Odd ratios (95% CI)	*P value*	Odd ratios (95% CI)	*P value*
Childhood Adversity	0.96 (0.79-1.17)	0.699	0.97 (0.69-1.36)	0.858	1.14 (0.88-1.46)	0.307	0.79 (0.51-1.22)	0.282	1.44 (1.10-1.85)	0.006	1.31 (0.82-2.11)	0.260	1.04 (0.83-1.29)	0.754	0.68 (0.46-1.00)	0.049
Emotional Abuse	1.33 (0.95-1.87)	0.087	1.37 (0.97-1.95)	0.077	1.05 (0.67-1.62)	0.745	1.04 (0.65-1.61)	0.862	1.50 (0.96-2.29)	0.057	1.29 (0.81-1.99)	0.270	1.30 (0.90-1.86)	0.136	1.29 (0.87-1.88)	0.194
Scarcity of food	0.91 (0.78-1.07)	0.291	0.90 (0.74-1.09)	0.270	1.23 (1.00-1.50)	0.041	1.21 (0.94-1.54)	0.138	1.19 (0.95-1.49)	0.099	1.05 (0.79-1.38)	0.752	1.07 (0.90-1.28)	0.42	1.11 (0.89-1.39)	0.340
Poor health status	0.97 (0.79-1.20)	0.875	0.97 (0.76-1.23)	0.806	1.11 (0.84-1.46)	0.404	1.15 (0.84-1.56)	0.377	1.38 (1.03-1.83)	0.022	1.17 (0.84-1.61)	0.351	1.08 (0.85-1.37)	0.512	1.22 (0.93-1.60)	0.148
Childhood maltreatment	0.96 (0.80-1.16)	0.749	0.98 (0.78-1.23)	0.884	1.13 (0.88-1.43)	0.301	1.15 (0.86-1.54)	0.342	1.07 (0.82-1.39)	0.550	0.90 (0.64-1.26)	0.555	1.18 (0.96-1.45)	0.095	1.24 (0.96-1.60)	0.096
Childhood migration	1.34 (0.87-2.09)	0.182	1.35 (0.89-2.06)	0.166	1.43 (0.84-2.36)	0.134	1.46 (0.87-2.35)	0.136	1.14 (0.60-2.02)	0.653	0.97 (0.52-1.67)	0.91	1.28 (0.80-2.01)	0.291	1.44 (0.90-2.24)	0.117

Notes: Adjusted and unadjusted odds ratios, 95% CI, to determine the association between childhood adversity and disease in adulthood (adjusting for the effect of age, gender, ethnic group, and low childhood SEP).

Supplementary Table 4. Associations of cumulative childhood adversity and cardiometabolic disease in later adulthood.

This Table suggests a significant gender interaction in the association between ACEs and the risk of cardiovascular disease. This indicates that the risk of CVD is not the same for men and women, and it is significantly higher for women than for men, suggesting that the impacts of ACEs on cardiovascular disease is influenced by gender.

	**Outcomes**							
	**Cardiovascular disease**		**Diabetes**		**Hypertension**		**Obesity**	
	**ORs (95% CI)**	***P*- value**	**ORs (95% CI)**	***P*- value**	**ORs (95% CI)**	***P*- value**	**ORs (95% CI)**	***P*- value**
**Full Sample**								
Intercept	0.12 (0.11 – 0.13)	**<0.001**	0.18 (0.17 – 0.20)	**<0.001**	0.97 (0.92 – 1.02)	0.220	0.54 (0.51 – 0.57)	**<0.001**
ACEs 1	1.20 (1.07 – 1.35)	**0.001**	1.02 (0.92 – 1.13)	0.688	1.14 (1.06 – 1.22)	**<0.001**	0.89 (0.82 – 0.96)	**0.002**
ACEs 2	1.46 (1.28 – 1.65)	**<0.001**	1.13 (1.00 – 1.26)	**0.041**	1.21 (1.12 – 1.32)	**<0.001**	0.89 (0.81 – 0.97)	**0.008**
ACEs 3	1.79 (1.53 – 2.10)	**<0.001**	1.29 (1.11 – 1.49)	**0.001**	1.18 (1.05 – 1.32)	**0.005**	1.00 (0.88 – 1.12)	0.942
ACEs 4+	1.81 (1.39 – 2.32)	**<0.001**	1.39 (1.09 – 1.76)	**0.007**	1.33 (1.10 – 1.62)	**0.003**	0.92 (0.75 – 1.12)	0.420
R^2 Tjur	0.004		0.001		0.002		0.001	
**Stratified by gender**								
Men								
ACEs 1	1.08 (0.92 – 1.28)	0.345	0.98 (0.83 – 1.15)	0.766	1.14 (1.02 – 1.27)	**0.021**	0.87 (0.77 – 0.98)	0.019
ACEs 2	1.18 (0.97 – 1.43)	0.100	1.13 (0.94 – 1.35)	0.207	1.21 (1.07 – 1.38)	**0.003**	0.85 (0.73 – 0.98)	0.024
ACEs 3	1.26 (0.98 – 1.61)	0.069	1.31 (1.04 – 1.65)	**0.023**	1.20 (1.02 – 1.42)	**0.032**	0.95 (0.79 – 1.14)	0.590
ACEs 4+	1.29 (0.83 – 1.93)	0.233	1.34 (0.89 – 1.95)	0.147	1.53 (1.15 – 2.05)	**0.004**	0.97 (0.70 – 1.33)	0.861
R^2 Tjur	0.001		0.001		0.002			
Women								
ACEs 1	1.31 (1.13 – 1.53)	**0.001**	1.03 (0.91 – 1.17)	0.616	1.11 (1.01 – 1.23)	**0.031**	0.88 (0.79 – 0.97)	**0.009**
ACEs 2	1.72 (1.45 – 2.04)	**<0.001**	1.11 (0.96 – 1.29)	0.156	1.19 (1.06 – 1.33)	**0.003**	0.89 (0.79 – 1.00)	**0.046**
ACEs 3	2.35 (1.91 – 2.89)	**<0.001**	1.27 (1.05 – 1.54)	**0.015**	1.16 (1.00 – 1.36)	0.057	1.03 (0.88 – 1.20)	0.729
ACEs 4+	2.28 (1.64 – 3.14)	**<0.001**	1.40 (1.03 – 1.88)	**0.028**	1.17 (0.91 – 1.51)	0.223	0.86 (0.67 – 1.12)	0.270
R^2 Tjur	0.009		0.001		0.001		0.001	
**with Interaction**								
ACEs 1*women	1.21 (0.96 – 1.52)	0.099	1.06 (0.86 – 1.30)	0.586	0.98 (0.85 – 1.13)	0.784	1.01 (0.87 – 1.18)	0.870
ACEs 2*women	1.46 (1.13 – 1.89)	**0.004**	0.99 (0.78 – 1.25)	0.918	0.98 (0.83 – 1.16)	0.813	1.05 (0.87 – 1.26)	0.615
ACEs 3*women	1.87 (1.35 – 2.59)	**<0.001**	0.97 (0.72 – 1.31)	0.842	0.97 (0.77 – 1.22)	0.782	1.08 (0.85 – 1.38)	0.525
ACEs 4+*women	1.77 (1.05 – 3.04)	**0.034**	1.05 (0.64 – 1.73)	0.852	0.76 (0.52 – 1.13)	0.172	0.89 (0.59 – 1.35)	0.578
Gender: women	0.86 (0.72 – 1.03)	0.100	1.40 (1.20 – 1.63)	**<0.001**	1.71 (1.53 – 1.91)	**<0.001**	1.66 (1.48 – 1.87)	<0.001
R^2 Tjur	0.006		0.005		0.018		0.015	

Note: Odds Ratios (ORs) and 95% confidence intervals (CI) are from logistic regressions of ACEs score on cardiometabolic disease.
